# ADC值与肺癌组织学类型及分化程度的相关性研究

**DOI:** 10.3779/j.issn.1009-3419.2012.11.01

**Published:** 2012-11-20

**Authors:** 菲 李, 铁链 于, 伟栋 李, 翀 张, 阳 曹, 大同 苏, 颖 王, 东 李

**Affiliations:** 1 300052 天津，天津医科大学总医院放射科 Department of Radiology, Tianjin Medical University General Hospital, Tianjin 300052, China; 2 221002 徐州，徐州医学院附属医院放射科 Department of Radiology, Affiliated Hospital of Xuzhou Medical College, Xuzhou 221002, China

**Keywords:** 肺肿瘤, 扩散加权成像, 表观扩散系数, 磁共振成像, Lung neoplasms, Diffusion-weighted imaging, Apparent diffusion coefficient, Magnetic resonance imaging

## Abstract

**背景与目的:**

肺癌的组织学类型和分化程度对于评估肿瘤的生物学行为、预后及选择治疗方式具有重要意义。本研究旨在探讨表观扩散系数（apparent diffusion coefficient, ADC）与肺癌组织学类型和分化程度的相关性。

**方法:**

符合纳入标准的连续肺癌患者115例，行DWI检查（b=500 s/mm^2^）并测量病变的ADC值，用*t*检验及单因素方差分析分析不同组织学类型及不同分化程度间ADC值的差异，用*Spearman*相关系数分析ADC值与不同分化程度之间的相关性。

**结果:**

小细胞肺癌（small cell lung cancer, SCLC）与非小细胞肺癌（non-small cell lung cancer, NSCLC）的ADC值差异有统计学意义（*P*=0.017），且SCLC与鳞癌、腺癌的ADC值差异均有统计学意义（*P*分别为0.011、0.001）。肺癌不同分化程度间的ADC值差异有统计学意义（*P*=0.003），且ADC值与病变不同分化程度存在相关性（*r_s_*=-0.272, *P*=0.003）。

**结论:**

ADC值对术前判断肺癌的组织学类型及分化程度有一定意义：小细胞肺癌的ADC值低，分化程度低的肿瘤的ADC值低。

## Abstract (en)

**Background and objective:**

The histologic type and grade of lung cancer are significant in assessing the biological behaviour, prognosis and therapeutic regimen. This study investigates the correlation of apparent diffusion coefficient (ADC) with histologic type and grade of lung cancer.

**Methods:**

A total of 115 patients pathologically diagnosed with lung cancer were enrolled in this study. Magnetic resonance (MR)-DWI with a diffusion factor of 500 s/mm^2^ was performed and the ADC values of lesions were measured. The ADC values among the different histologic types and grades were compared with the values obtained using *t*-test and one-way *ANOVA*. The correlation between ADC values and the histologic grades was further evaluated with *Spearman* correlation coefficient.

**Results:**

With a b value of 500 s/mm^2^, the ADC values significantly differed between small cell lung cancer (SCLC) and non-small cell lung cancer (NSCLC) (*P*=0.017). The ADC values of SCLC were significantly different from those of squamous cell carcinoma and adenocarcinoma (*P*=0.011, 0.001, respectively). A significant difference was observed in the ADC values among the different histologic grades of lung cancer (*P*=0.003), and the ADC value was correlated with the histologic grades (*r_s_*=-0.272, *P*=0.003).

**Conclusion:**

ADC value is significant for judgement of histologic type and grade of lung cancer before surgical operation: SCLC has a low ADC value, and ADC value is low for the tumor with poorly-differentiated grade.

肺癌发病率在世界上高居恶性肿瘤首位，其组织学类型和分化程度对于评估肿瘤的生物学行为、预后及选择治疗方式具有重要意义。CT主要通过肿瘤形态学表现和血供特征评价肺癌，对肿瘤内部病理状态的评估存在局限性。磁共振扩散加权成像（diffusion-weighted imaging, DWI）是目前唯一能够活体检测水分子微观运动的功能成像技术，在临床上已体现出较高的应用价值。近年来，随着回波平面成像（echo planar imaging, EPI）技术的迅速发展，以及多通道线圈、并行采集技术和运动伪影抑制等技术的研发和应用，使得DWI在胸部的应用越来越广泛。DWI对肺部病变，特别是对肺癌的诊断与鉴别诊断、分期和疗效评估等方面的价值越来越多地受到关注^[1-5]^。本研究旨在探讨表观扩散系数（apparent diffusion coefficient, ADC）与肺癌组织学类型及分化程度的关系。

## 1 资料与方法

### 1.1 研究对象

2009年6月-2011年10月就诊于天津医科大学总医院肺外科、胸外科、呼吸科，并符合纳入标准的连续肺癌患者115例。纳入标准：①病理学（手术、支气管镜活检或穿刺活检）证实为肺癌；②接受胸部CT和MRI检查，且两种检查间隔时间 < 1周；③CT和MRI均可以显示肺癌原发病灶；④影像学检查前未接受任何放疗和化疗。

### 1.2 检查方法

MRI采用GE signa HDxt 3.0T磁共振扫描仪和Torsopa相控阵表面线圈，检查序列包括：①常规MR平扫：快速弛豫快速自旋回波脉冲序列（fast relaxation fast spin echo, FRFSE）T2加权成像（T2 weighted imaging）（FRFSE T2WI），呼吸触发和心电触发（R波触发），TR/TE（8, 000-8, 571）ms/（86-96）ms，层厚/间隔4.0 mm/1.0 mm，NEX 2，ETL 20，FOV 42 cm，矩阵256×160，并于轴位T2WI施加预饱和脂肪抑制技术得到轴位压脂T2WI；②DWI：扫描前先行ASSET校准序列扫描，然后采用单次激发自旋回波-回波平面成像序列（spin echo-echo planar imaging, SE-EPI）行轴位DWI扫描，在自由呼吸状态下采集图像，b值取0 s/mm^2^、500 s/mm^2^，同时在X、Y、Z轴三个方向上施加敏感梯度脉冲。

CT采用64排螺旋CT扫描仪（GE LightSpeed V CT XT），扫描参数为120 kV、300 mA、5 mm层厚，FOV 360 mm，矩阵512×512。

### 1.3 图像后处理及数据测量

使用AW 4.3工作站的Functool 4.5.5软件包对图像进行后处理，获得病变的DWI图、ADC图。参考T2WI及脂肪抑制T2WI图，于DWI图、ADC图选择病灶信号强度最大且最均匀的层面设置圆形或椭圆形的ROI（region of interest），测量病变区ADC值。所取ROI包括病灶最大径线的60%以上，并尽可能包括最大信号强度中心区域，避开病变边缘和肉眼可辨的坏死区。所有病灶的ADC值均测量3次，计算其平均值作为最终测量值。

### 1.4 统计分析方法

使用SPSS 17.0统计分析软件。采用两独立样本*t*检验及单因素方差分析分析不同组织学类型及不同分化程度肿瘤ADC值的差异；采用两变量间相关分析计算*Spearman*相关系数，分析ADC值与分化程度之间的相关性。*P* < 0.05为差异有统计学意义。

## 2 结果

### 2.1 组织病理学分析和分化程度

115例肺癌病人年龄38岁-85岁，平均（59.45±8.65）岁，男71例，女44例。共发现肺癌病灶115个，病灶最大径线范围1.4 cm-12.2 cm，平均（6.2±2.4）cm。组织学结果：鳞癌44例（38.3%），腺癌43例（37.4%），小细胞肺癌（small cell lung cancer, SCLC）27例（23.5%），大细胞肺癌1例（0.8%）。因大细胞肺癌仅为1例，故未进行进一步的统计学分析，本研究实际统计的病例数和病灶数为114例。其中，高分化组24例（21%）、中分化组32例（28%）、低分化组58例（51%）。

### 2.2 不同组织学类型ADC值比较

图 1与图 2分别为SCLC和中分化鳞癌各1例患者的代表图。不同组织学类型的ADC值比较见图 3、图 4。SCLC的ADC值小于非小细胞肺癌（non-small cell lung cancer, NSCLC），且差异有统计学意义（*t*=-3.364, *P*=0.017）。鳞癌、腺癌、SCLC的ADC值差异也有统计学意义（*F*=6.231, *P*=0.003），两两组间比较显示SCLC与鳞癌、腺癌的ADC值差异均有统计学意义（*P*分别为0.011、0.001），而鳞癌和腺癌的ADC值差异无统计学意义（*P*=0.29）。

**1:**
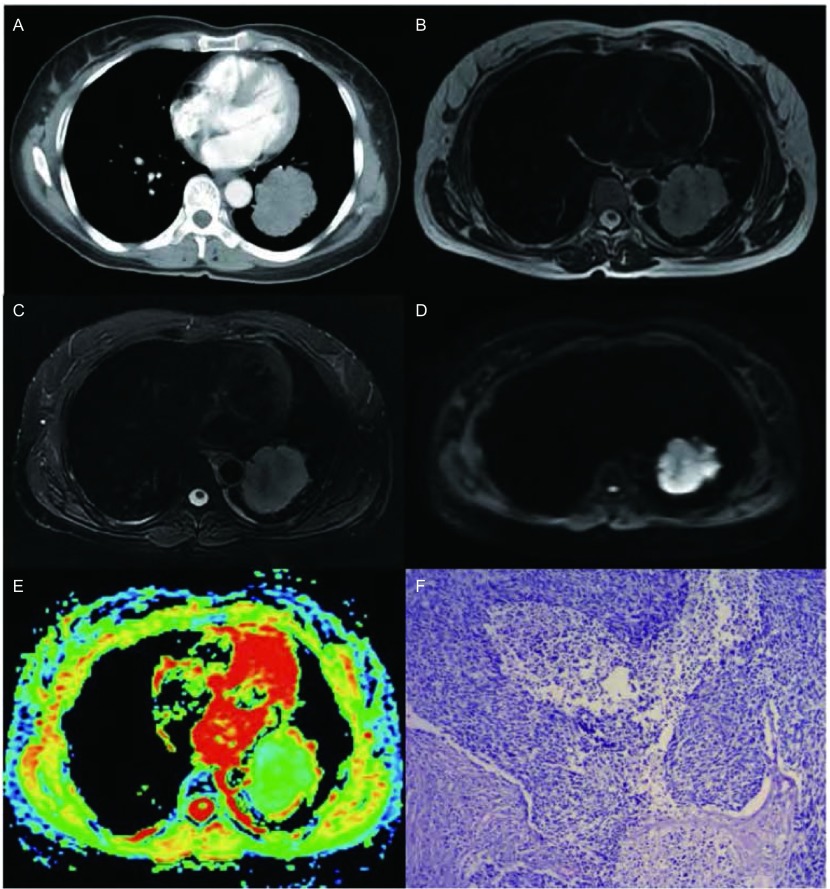
女，53岁，小细胞肺癌。A-E：增强CT、T2WI、T2WI抑脂像、DWI图及ADC图（b=500 s/mm^2^）。增强CT图显示左肺下叶不规则软组织肿块，于T2WI及T2WI抑脂像上呈高信号，于DWI图上呈明显高信号，于ADC图上以绿色区域为主，ADC值为1.10；F：HE染色病理切片（×100）。 A 53-year-old woman with small cell lung cancer (SCLC). A-E: Contrast enhanced CT, T2WI, fat suppressed T2WI, DWI and apparent diffusion coefficient (ADC) map(b=500 s/mm^2^). The contrast enhanced CT shows a tumor in inferior lobe of left lung, and it displays hyperintense in T2WI and fat suppressed T2WI. On DWI, the tumor was obvious hyperintense. On ADC map, the tumor was depicted as an area of green with the ADC value of 1.10; F: Histologic sections (100×magnification, H & E staining).

**2:**
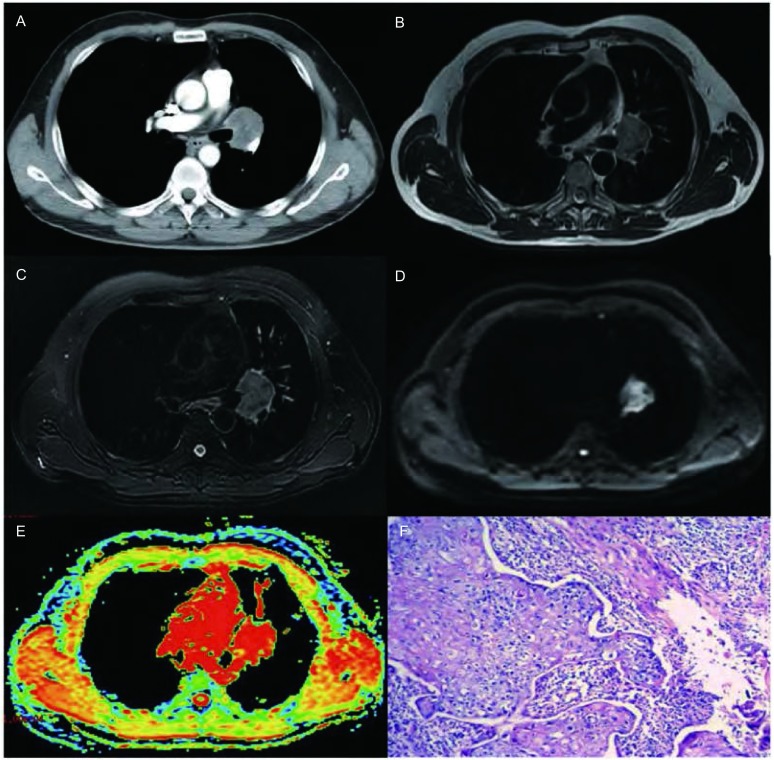
男，51岁，中分化鳞癌。A-E：增强CT、T2WI、T2WI抑脂像、DWI图及ADC图（b=500 s/mm^2^）。增强CT图显示左肺门软组织肿块，于T2WI及T2WI抑脂像上呈高信号，于DWI图上呈不均匀高信号，于ADC图上以红色区域为主，ADC值为1.67；F：HE染色病理切片（×100）。 A 51-year-old man with moderately differentiated squamous cell carcinoma. A-E:Contrast enhanced CT, T2WI, fat suppressed T2WI, DWI and ADC map (b=500 s/mm^2^). The contrast enhanced CT shows a tumor in hilum of left lung, and it displays hyperintense in T2WI and fat suppressed T2WI. On DWI, the tumor was inhomogenous hyperintense. On ADC map, the tumor was depicted as an area of red with the ADC value of 1.67; F: histologic sections (100×magnification, H & E staining).

**3:**
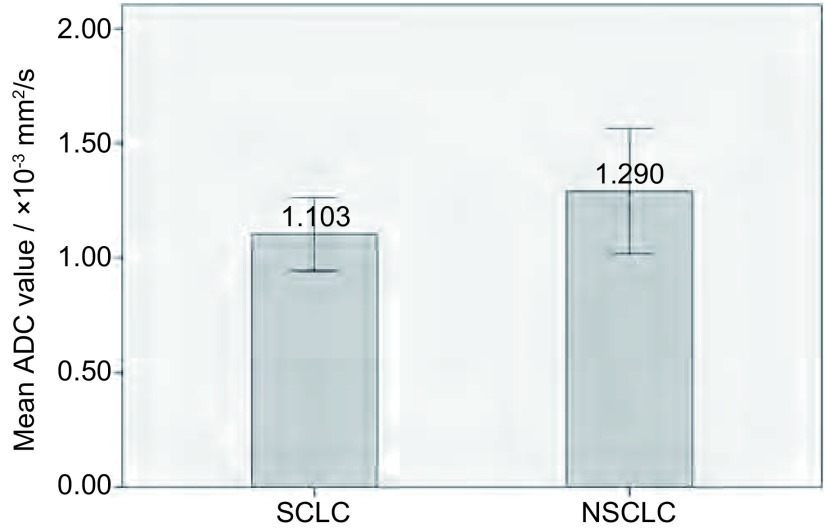
SCLC与NSCLC的ADC值比较 Comparison of mean ADC values of tumors between the SCLC and non-small cell lung cancer (NSCLC)

**4:**
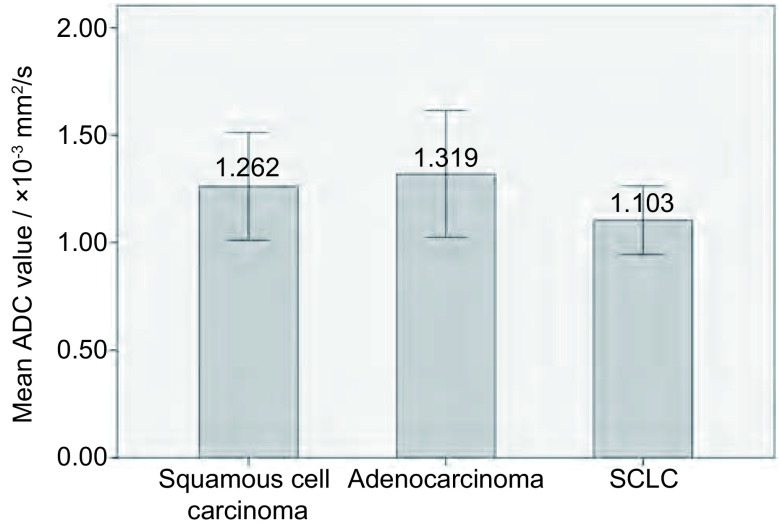
不同组织学类型间ADC值比较 Comparison of mean ADC values of tumors among different histologic types

### 2.3 不同分化程度ADC值比较

不同分化程度肺癌间的ADC值差异有统计学意义（*F*=6.161, *P*=0.003）（图 5），两两组间比较显示低分化组与高分化组的ADC值差异有统计学意义（*P*=0.001），余两组间无统计学差异。*Spearman*相关分析表明，ADC值与肺癌分化程度存在相关性（*r_s_*=-0.272, *P*=0.003）。

**5:**
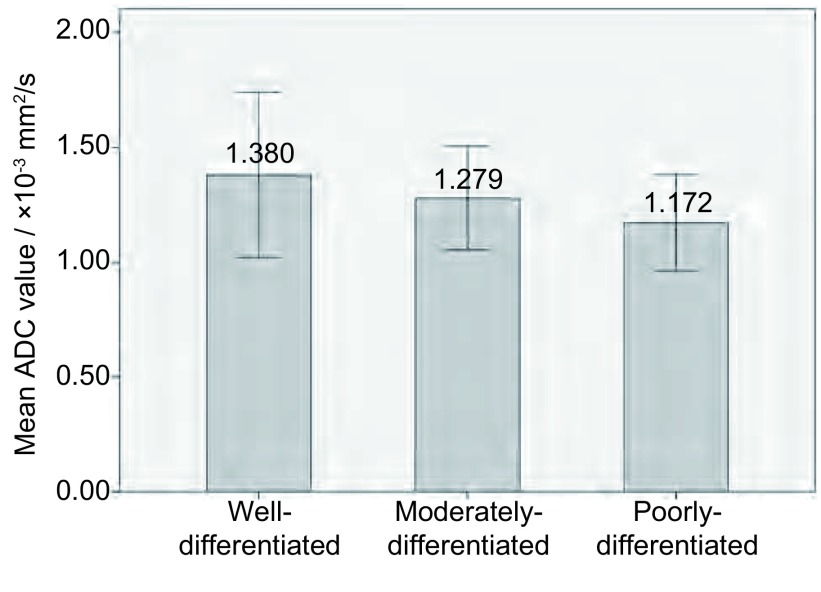
肺癌不同分化程度间ADC值比较 Comparison of mean ADC values among different differentiation grades of lung cancer

## 3 讨论

DWI能够在活体检测组织内水分子的扩散运动，间接反映组织微观结构和功能状态的改变，为疾病的诊断和鉴别诊断提供分子水平的信息。ADC值用来描述在活体扩散成像上所观察到的表观扩散现象，实现了DWI的量化分析。ADC值=ln（SI_低_/SI_高_）/（b_高_-b_低_），SI_低_及SI_高_分别代表用低b值及高b值所得到的DWI图像的信号强度。本研究选择b值为0 s/mm^2^、500 s/mm^2^，既能获得较佳的图像信噪比和对比噪声比，且能较准确反映组织扩散的真实性。DWI在肺内良、恶性实性病变的鉴别中表现出较高的临床应用价值^[2, 3]^。然而，对于肺癌而言，其组织学类型及分化程度是重要的预后因子，ADC值与肺癌组织学类型及分化程度的相关性研究具有重要的临床意义。

本研究结果表明，SCLC的ADC值小于NSCLC（*P*=0.017），且SCLC与鳞癌、腺癌的ADC值差异均有统计学意义（*P*分别为0.011、0.001）。宫颈癌、前列腺癌、乳腺癌等其它部位肿瘤的DWI研究表明，ADC值和肿瘤细胞密度呈负相关^[5-10]^。在组织病理学上，SCLC通常由小圆形或卵圆形的癌细胞组成，类似淋巴细胞，呈弥漫或片状分布，细胞密度高，细胞外间隙小^[11]^，组织中的水分子扩散运动受限，因此病变信号在DWI上明显升高，ADC值明显降低。Liu等^[3]^、Razek等^[5]^的研究也表明SCLC与NSCLC的ADC值间的差异。因此，肺癌ADC值的测量有可能为SCLC和NSCLC的鉴别提供一个新途径，对临床治疗方案的制定提供一定的参考。

虽然本研究腺癌组和鳞癌组的ADC值差异没有统计学意义，但腺癌组ADC值有大于鳞癌组的趋势。Matoba等^[1]^发现腺癌的ADC值明显高于鳞癌及大细胞肺癌。组织的ADC值受细胞密度影响，而坏死以增加水分子弥散为特征，这意味着ADC值的改变在组织切片及T2加权像改变之前被观察到。因此，组织内坏死的程度及坏死之前微结构的变化，都有可能是影响ADC的因素。因此，未来除扩大病例样本量来进一步证实不同组织学类型间ADC值的差异外，还可以对组织切片及DWI图像进行微观精确的对照分析，来进一步明确肺癌ADC值的影响因素。

本研究发现高、低分化肺癌ADC值的差异有统计学意义。Razek等^[5]^研究表明低分化肺癌的ADC值明显低于高分化及中分化肺癌，Matoba等^[1]^发现高分化腺癌的ADC值明显高于中低分化鳞癌及低分化腺癌。虽然上述各研究结果发现有统计学差异的组别不同，但是这些研究结果均表明不同分化程度肺癌的ADC值有差异。本研究结果中，虽然高、中分化组及中、低分化组之间的ADC值差异无统计学意义，但是随着分化程度的降低，ADC值有逐渐降低的趋势，而且*Spearman*相关分析表明ADC值与肺癌分化程度存在相关性（*r_s_*=-0.272, *P*=0.003），即肿瘤级别越高，分化程度越低，ADC值越低；反之，肿瘤级别越低，分化程度越高，ADC值越高。这可能是由于肺癌不同分化程度间肿瘤细胞的排列方式、细胞密度、周围间质均存在差异，组织分化程度越低，细胞数量增多、排列致密、胞外间隙减小致细胞外水分子运动减慢，且肿瘤细胞内胞核增大、细胞器增多、胞浆减少致细胞内水分子运动减慢，故ADC值也越低。另有研究^[12]^表明，高分化的肿瘤细胞有丝分裂速度较慢，以管状生长模式生长，而低分化肿瘤细胞中，只有 < 10%的病变以管状方式排列，其高度的多形核及较快的有丝分裂也是促使ADC值降低的原因。ADC值与乳腺癌、前列腺癌及直肠癌肿瘤级别的相关性研究也有相似的发现^[12-14]^。

综上所述，ADC值对术前判断肺癌组织学类型及分化程度有一定的提示意义，对于肺癌临床治疗方式的选择及预后有一定的指向性。
